# Peer Review of “COVID-19 Pneumonia Diagnosis Using Medical Images: Deep Learning-Based Transfer Learning Approach”

**DOI:** 10.2196/83231

**Published:** 2025-09-26

**Authors:** Sunny Chi Lik Au

**Affiliations:** 1 Tung Wah Eastern Hospital, So Kon Po, China (Hong Kong)

**Keywords:** computer vision, COVID-19 pneumonia diagnosis, deep learning, transfer learning, medical imaging analysis

*This is the peer-review report for “COVID-19 Pneumonia Diagnosis Using Medical Images: Deep Learning-Based Transfer Learning Approach.”*

## Round 1 Review

### General Comments

This paper [1] focused on the use of artificial intelligence (AI), in particular convolutional neural networks (CNNs) for detection of COVID-19 infections in radiological imaging. The study uses a substantial dataset of over 6000 images, which enhances the reliability of the results and supports robust model training and evaluation. Leveraging well-known CNNs such as VGG16, VGG19, and ResNet-50 demonstrates a practical application of transfer learning, a widely accepted technique in deep learning for medical imaging tasks.

However, in the Background and Introduction sections, the authors focused on the importance of rapid and early diagnosis of COVID-19, thus the demand for AI CNNs for diagnosis (“traditional diagnostic methods, such as serologic tests, have limitations, including low sensitivity and longer processing times”), yet this could be achieved easily nowadays with lateral flow devices or rapid antigen tests. Using computed tomography or X-rays to screen COVID-19 is far too expensive and time consuming compared to lateral flow devices or rapid antigen tests.

I believe the author was referring to the use of AI CNNs to differentiate COVID-19 pneumonia from other causes of pneumonia. Diagnosis of COVID-19 infection (which is usually mild and self-limiting) is totally different from COVID-19 pneumonia (which might require hospitalization and medical interventions). The authors might consider changing the title of the manuscript to “COVID 19 Pneumonia Diagnosis Analysis Using Transfer Learning–Deep Learning.” Similarly, for section 3.1, “COVID-19 Pneumonia Diagnosis Using Deep Learning” would be more appropriate than “COVID-19 Diagnosis Using Deep Learning.”

In addition, the Related Work section is brief and lacks depth. It does not sufficiently review existing medical studies on deep learning for COVID-19 pneumonia diagnosis, making it less comprehensive.

### Specific Comments

#### Major Comments

1. Since this is a medical journal, medical terms are encouraged, for example, *anosmia* to replace *loss of smell*; *ageusia* to replace *loss of taste*.

2. Quite a significant number of references were not medical related, but related to AI or computer science. I would suggest the authors visit PubMed to search for more medical-related references. I cannot suggest any particular references to avoid conflicts of interest with certain groups of authors and to avoid self-citation.

3. The author detailed the AI CNN mechanism, yet the features of computed tomography or X-rays that were focused on were not mentioned. Was ground glass appearance the main target, or was it other features like cavitation, extent of lung involvement, or superior location? It would be more valid to evaluate various features targeted by the AI, instead of mentioning how it works.

#### Minor Comments

4. The author cited many different online references, yet the links or URLs were not available for readers to refer to. I would suggest adding the cited reference sources back for reviewers to assess the appropriateness of the citation, such as references 26 to 28, and for the benefit of readers. For example, reference 9 is not searchable on the internet.

5. In section 1.1, “At that point, there have been 98 confirmed cases and no reported deaths in 18 countries outside China...” Please add a reference citation for this factual statement.

6. In section 1.1, “As of 28 April 2020, 63% of worldwide mortality from the virus was from the Region...” Please clarify the “Region.”

7. In section 1.3, “Motivation to try to COVID-19 Diagnosis,” the English could be further polished, for example, “Motivation-to-try to COVID-19 Diagnosis” or “Motivation to try towards COVID-19 Diagnosis.”

8. *Computed tomography*, instead of *computer tomography*, is the proper term in section 3.1.

9. Abbreviations need not be spelled out again after their first use in the main text. For example, “computer tomography (CT)” in section 3.1 can just be “CT,” since CT has been defined already.

10. Please be consistent with reference citation formatting; various formats are used in the reference list, for example, “[20] Z. Wu et al Unsupervised feature learning via non-parametric instance discrimination. In Proceedings of the IEEE Conference on Computer Vision and Pattern Recognition, pages 3733‐3742, 2018”; “[24] Md. Islam, F. Karray, R. Alhajj, and J. Zeng. A review on deep learning techniques for the diagnosis of novel coronavirus (covid-19). IEEE Access, vol. 9, pp. 30551‐30572, 2021. doi: 10.1109/ACCESS.2021.3058537”; and “[29] Mohammed K. Hassan, Ali I. El Desouky, Sally M. Elghamrawy, and Amany M. Sarhan. A hybrid real-time remote monitoring framework with nb-woa algorithm for patients with chronic diseases. https://doi.org/10.1016/j.future.2018.10.021, 2019. Future Generation Computer Systems, Volume 93, Pages 77‐95, ISSN 0167‐739X.”

11. To further improve the manuscript, please consider adding figures or tables showing the appearance of COVID-19 versus normal samples. Add to the Limitations section a discussion of potential biases (eg, dataset origin) or generalizability issues (eg, applicability to new variants) to demonstrate critical reflection

## Round 2 Review

### General Comments

Thanks for revising the manuscript according to the review comments. Most of my concerns are now addressed.

### Specific Comments

#### Major Comments

1. Some parts of the manuscript used extensive bulleted lists; paragraphs should be used in the manuscript’s main text. If the author deems bullet points more appropriate for the content, the author could format lists as tables.

## Abbreviations

AI: artificial intelligence
CNN: convolutional neural network
